# The role of unexplained high serum alpha-fetoprotein (AFP) and human chorionic gonadotropin (hCG) levels in the second trimester to determine poor obstetric outcomes

**DOI:** 10.4274/tjod.00922

**Published:** 2014-09-15

**Authors:** Hümeyra Öztürk, Salim Erkaya, Sibel Altınbaş, Burak Karadağ, Nazan Vanlı Tonyalı, Demet Özkan

**Affiliations:** 1 Sincan State Hospital Ministry of Health, Clinic of Obstetrics and Gynecology, Ankara, Turkey; 2 Zekai Tahir Burak Women’s Health Education and Research Hospital, Ankara, Turkey; 3 Kastamonu Hacettepe University Faculty of Medicine, Department of Obstetrics and Gynecology, Kastamonu, Turkey; 4 Ankara Teaching and Research Hospital Ministry of Health, Clinic of Obstetrics and Gynecology, Ankara, Turkey; 5 Aksaray State Hospital Ministry of Health, Clinic of Obstetrics and Gynecology, Aksaray, Turkey

**Keywords:** Serum screening test, alpha-fetoprotein, human chorionic gonadotropin, poor obstetric outcome

## Abstract

**Objective:**

To investigate the relationship between gestational complications and high levels of maternal serum alfa-fetoprotein (MSAFP) and/or beta human chorionic gonadotropin (hCG) and to determine whether these markers are effective predictors of poor pregnancy outcomes.

**Materials and Methods:**

In this study, we enrolled a total of 679 women at 15-20 gestational weeks with MSAFP and hCG below or above 2.0 multiples of the median (MoM); of those, 200 women with normal MSAFP and hCG MoM formed the control group. Pre-eclampsia, intrauterine growth retardation (IUGR), preterm labor, preterm delivery, placental abruption, placenta previa, placenta accreta, preterm premature rupture of the membranes (PPROM), intrauterine fetal death, as well as neonatal and perinatal morbidity rates were evaluated.

**Results:**

A significant relationship was found between adverse pregnancy outcomes and abnormal elevation of hCG and AFP levels in the second trimester. In cases of isolated elevation of hCG, preeclampsia and preterm labor/spontaneous preterm birth rate were slightly higher than in the control group (p=0.043, p=0.015), while IUGR, PPROM, placental abruption, and intrauterine fetal death rates were all similar (p=0.063, p=0.318, p=1.00, p=0.556).

In case having an elevation in both markers, increased rate of obstetric complications have been observed. A significant relationship was found between the high levels of maternal serum AFP and hCG MoM and poor pregnancy outcomes like preeclampsia, IUGR, PPROM, intrauterine fetal death (p=0.003, p=0.001, p=0.040, p=0.006).

**Conclusion:**

To our knowledge, up to now, no definitive follow-up and treatment protocols have been established for patients at increased risk. In light of these findings, it is recommended to inform and educate patients about the most likely signs and symptoms of complications, to make more often antenatal visits, to perform more frequent ultrasound examination (fetal growth, AFI, etc.), NST, arterial/venous doppler, biophysical profile, and cervical length measurements in high-risk group.

## INTRODUCTION

Diagnosing many complications related to gestation, such as intrauterine growth retardation, preeclampsia, placental abruption in early gestational weeks by several safe tests is of vital importance in terms of reducing morbidity and mortality rates. Biochemical indicators started to be used for researching fetal genetic disorders in 1980s and 90s. In the beginning, these tests that were started to be used only for researching the pregnant under risk in terms of neural tube defects, but subsequently they were started to be used also for researching other anatomical malformations, aneuploidy and third trimester complications later^(1,2)^.

Upon understanding that median values of alpha feto protein (AFP), unconjugated estriol (uE3), human chorionic gonadotropin (hCG) concentrations of the serums of the pregnants who have normal fetus in the sixteenth gestational week are different from the median value of trisomy 21 fetus carrying pregnants, triple test run was suggested considering that these hormones can be used in screening the high risk group. Besides though test results were not in risk area for trisomy, it was detected that structural fetal anomalies such as open neural tube defect (NTD), abdominal wall defect and placental anomalies were accompanied by high AFP and\or high hCG levels.

AFP and\or hCG levels can be found high in approximately 1% of the pregnant women without gestational age estimating mistake, structural or a chromosomal anomaly, or multiple pregnancy^(3)^.

The relation between unexplained high AFP and\or hCG and adverse antenatal outcomes has been recognized in the last 20 years^(4,5)^. It was shown that unexplained high AFP may be related with preterm labor, IUGR, preeclampsia and fetal death^(6,7)^. On the other hand it was stated that elevated maternal hCG in the 2. trimester was related with preeclampsia^(8,9)^ and increased fetal death rates^(7,10,11)^.

The purpose of this study is to evaluate the relationship between gestational complications like preterm labor, preterm birth, PPROM, stillbirth, IUGR, preeclampsia and high MSAFP and/or beta hCG levels. Also we aimed to detect whether these markers are effective predictors of adverse pregnancy outcomes or not.

## MATERIALS AND METHODS

Between June 2009 - November 2010, total 500 pregnant women who had applied to Prenatal Clinic of Etlik Zübeyde Hanım Education and Research Hospital for triple test between 15 to 20 gestational weeks were enrolled in this study. The patients were divided into 4 groups considering AFP and HCG MoM values. While group 1, consisting of 200 patients with normal ranges (0.5-2.0 MoM) of both AFP and HCG MoM values, is determined as control group, group 2, consisting of 100 patients with high HCG values (above 2.0 MoM) and AFP values within normal ranges, group 3 consisting 100 patients with HCG values within normal ranges and high AFP values (above 2.0 MoM) and group 4 consisting 100 patients with both AFP and HCG MoM values above are identified as study groups.

All cases were informed about the screening test and detailed consent forms were obtained. The study protocol was approved by the local ethical committee.

Inclusion criteria were stated as follows:

1) Single live pregnancy

2) Gestational age between 15-20 week

3) Regularly antenatal follow-up

Exclusion criteria were:

1) Discordant gestational age according to first trimester ultrasounds

2) Multiple pregnancy

3) Lack of antenatal follow-up

4) Fetoplacental and chromosomal anomaly

5) Insulin-dependent diabetes mellitus

6) Over 35 years old

Research was started with 679 pregnants and 12 patients due to diabetes with insulin, 6 patients due to fetoplacental and chromosomal anomaly, and 161 patients due to not being screened were taken out of the research.

After it was confirmed that current pregnancy was matching with the last menstrual period (LMP) with the help of ultrasonography (USG) measurement, AFP (ng\ml) and hCG (mIU\ml) laboratory assessment were measured with Irma CT irrigation method via E170 brand device by using Roche brand AFP and hCG kits. Estriol (ng\ml) was measured with the RiA method by using Roche brand E3 kit.

MoM rates were calculated by using stated pregnancy week, maternal age, sample obtaining date and AFP, hCG, uE3 rates for each patient. MoM rates were measured by dividing into median values of the gestational week on the date of sample obtaining. Cases with at least 2.0 MoM AFP rates were considered as positive test for neural tube defect. Patients with at least 1\250 down syndrome risk were considered positive for the screening test and were presented the option of invasive procedures. When the literature was analysed; some studies showed that pregnancy complications are increased when AFP and hCG values are more than 2 MoM and pregnancies with the higher values of AFP and hCG are terminated for fetal anomalies, so 2.0 MoM was taken as limit value.

With the laboratory results taken on basis, obstetric care were not changed by increasing specific fetal methods such as non-stress test or USG. Antepartum tests or additional USG were not performed out of the obstetric indications. Patients’ data was evaluated after delivery.

The relation between obstetric and neonatal pathologies (preeclampsia, intrauterine growth retardation (IUGR), preterm labor, PPROM, intrauterine fetal death, neonatal and perinatal morbidity) and high MSAFP and / or beta HCG levels were investigated.

### Statistical Analysis

Data analysis was carried out using SPSS (Statistical Program of Social Sciences) ver. 17.0 (SPSS Inc., Chicago, IL, USA). Continuous variables were expressed as mean, median, minimum and maximum, whereas percentages and frequencies were used for categorical variables. Groups were controlled in terms of conformity to normal distribution by graphical check and Shapiro Wilk test. Kruskall-Wallis variance analysis was performed for not normally distributing continuous variables and ANOVA was used for normally distributed continuous variables. Intergroup differences for categorical values were assessed with chi square test. Crosstabs and Roc curve were used for calculating sensitivity, specificity, positive predictive value and negative predictive value. The Pearson correlation analysis was used for determining the relations between perinatal outcomes and AFP, hCG and uE3. A p-value <0.05 was considered statistically significant.

## RESULTS

Demographic and obstetric characteristics of the patients were presented in Table 1. There was no significant differences for demographic and obstetric characteristics between the groups (p>0.05). According to the last menstruation period and ultrasonographic assessment, pregnancy weeks were similar.

In Table 2, the mean and standard deviation values of AFP, hCG and uE3 for all groups were presented.

When we analyze occurrence rates of maternal complications in group 1, 2, 3 and 4, the rates were determined as 8.5% (n=17), 25% (n=25), 21% (n=21) and 35% (n=35) respectively. Pregnancy complications were statistically significant higher in group 2, 3 and 4 when compared with control group (group 1) (p=0.001, p=0.002, p=0.001). Pregnancy-related complications and type of delivery of the groups were shown in Table 3.

Sensitivity of isolated elevation of hCG (group 2) in predicting pregnancy complications was calculated as 25% whereas specificity as 91.5%, positive predictive value as 59.52% and negative predictive value as 70.93%. Sensitivity of isolated elevation of AFP (group 3) in predicting pregnancy complications was calculated as 21% whereas specificity as 91.5%, positive predictive value as 55.26% and negative predictive value as 69.85%. And for the last sensitivity of both high levels of hCG and AFP (group 4) in predicting pregnancy complications was calculated as 35% whereas specificity as 91.5%, positive predictive value as 67.31% and negative predictive value as 73.79%. Odd’s ratios were determined for group 2, 3 and 4 as 3.56 (95% CI= 1.83-7.02), 2.86 (95% GA = 1.43-7.02) and 5.80 (95% CI= 3.04-11.04), respectively Table 4.

When cesarean indications were analysed, it was determined that the lowest cephalopelvic disproportion rate and the highest fetal distress and abnormal umbilical artery doppler rates were in group 4 while the rate of fetal macrosomia is the highest in group 2.

For the neonatal outcomes, it was seen that birth weight, pregnancy week of delivery and Apgar score were significantly lower in group 3 and 4 (p>0.001) when compared with group 1 and 2. Also, the neonatal intensive care need and neonatal death were significantly higher in group 4 when compared with the other groups.

## DISCUSSION

With the development of prenatal biochemical screening test programs, studies about relationship between increased AFP and hCG rates and adverse perinatal outcomes started to be published^(12)^.

In a prospective study conducted by Konachuk and friends, 35% of the pregnants with unexplained increased AFP level had at least one adverse perinatal outcome^(13)^. Similarly in many retrospective studies, it was found that in second trimester, increased hCG rates were in relation with increased antenatal complications^(9,14,15)^.

It is thought that adverse perinatal outcomes are associated with placental function disorders in patients with unexplained increase of maternal serum markers. Elevation of AFP was related to increased transition from feto-maternal circulation due to the placental feto-maternal surface damage^(16)^. Abnormally increased hCG levels are thought to have occured as a result of decreased placental perfusion related to low oxidation stemming, cytotrophoblasts’ abnormal placentation induced by hypoxia were shown in the histological studies conducted by Lieppman et al.^(15)^.

Brock et al. have stated that low birth weight (<2500 g) incidence is 10.7% when AFP level is above 2.3 MoM with no situation resulting this elevation such as twin pregnancy, NTD or fetal death, while 4.2% in the control group^(17)^.

Similarly, Wald et al. reported that an increased incidence of low birth weight, prematurity and perinatal death in pregnants with AFP level above 3 MoM^(18)^.

When Heinonen and his friends analyzed the relation between hCG rates above 2.0 MoM and maternal complications and adverse perinatal outcomes, they have concluded that above this value, preeclampsia, low birth weight, IUGR, velamentous umbilical cord insertion risks have increased but there has not been a statistical significant difference in preterm birth, fetal distress, fetal-perinatal death and newborn intensive care units admission rates compared to the control group^(19)^. Sorensen et al. have stated that there could be a high risk factor for preeclampsia when hCG increases.

In a retrospective study conducted by David et al., 28743 pregnant records have been analysed and it has been determined that in 2561 patients, hCG MoM rate was above 2 and that in these patients, preeclampsia incidence has increased. In spite of that, a relation between gestational diabetes, PPROM, IUGR and small (SGA) according to gestational age has not been determined^(20)^.

Persson and friends, in a research they have conducted on 10147 pregnants, have taken AFP cut-off rate as 2.3 MoM and have reported that 2.8-fold increase in low birth weight, 2-fold increase in preterm labor risk, 3-fold increase in perinatal death risk. Besides, in this research, 10-fold increase in placental abruption risk was reported^(21)^.

In a study, Milunsky and friends were investigated 13486 pregnant women and they reported that 4-fold increase in low birth weight risk, 3-fold increase in placental abruption risk, 8-fold increase in intrauterine fetal death risk and 2.3-fold increase in preeclampsia risk when AFP value was above 2 MoM^(22)^.

In another study Williams and friends compared obstetric complications of 201 patients with AFP MoM rate above 2.0 and 211 patients with AFP MoM rate below 2. Increase in preterm birth risk (OR=3.6), IUGR (OR=4), preeclampsia (OR=3.8), and placental abruption (OR=4.8) were determined and it was suggested that in these patients, placenta must be analysed carefully with USG^(23)^.

In the previous studies, it has been observed that, similarly to our research, obstetric complication rate has increased in more than one marker^(24,25)^. Dugoff and friends have reported that in first and second trimester fatal aneuploidy risk (FASTER) study, obstetric complication risks such as preterm birth, preeclampsia, fetal death have increased correspondingly with number of abnormal marker^(24)^.

After relation between unexplained increased AFP and\or hCG levels and antepartum complications in the second trimester, the question of “What can be done to increase the antenatal survival in pregnancy?” has been raised. In a study Huerta-Enochian and friends has shown that among the women with unexplained AFP rate early and frequent follow-ups to increase antenatal survival did not improve outcomes^(26)^.

However these results contradicts with other studies conducted by performing biophysical profile and umbilical artery doppler as their basis in which they reported increased fetal survival^(27,28)^.

Although there is a relationship between the abnormal maternal serum levels and poor obstetric outcomes, these tests remain only as a research tool due to their low sensitivity and positive predictive values. Therefore, they can not be used in clinical practice as screening test.

In our research, too, even though relationship with high AFP and\or hCG level and maternal complications have been found statistically significant (p=0.001), our sensitivity and positive predictive rates were low.

Even though some researchers have stated that adverse pregnancy outcomes would be decreased by performing serial ultrasound and uterine artery doppler measurements on pregnants with unexplained high maternal serum AFP and hCG rates, in a study conducted by Hamid and friends reported that such kind of an observation cannot make an improve in the outcomes^(29)^.

How early to start evaluation of the fetal well-being and to whom to apply serial tests with pregnants diagnosed with unexplained increased AFP and\or hCG are other questions. Van Rijn and friends have stated that, if there occurs a fetal death, it occurred approximately the 28^th^ week of gestation in women whose serum markers are normal, and it occurred approximately the 20^th^ week of gestation in women with increased AFP and hCG rates^(30)^.

Although our purpose has been to determine a distinctive limit value between the pregnants to be diagnosed with normal and complications for AFP and hCG, in our study especially the limit value we have accepted for AFP (for the reason that pregnancies having higher rates are ended with the diagnose of fetal anomaly) is low (2.0 MoM) and working with only one serum sample have effected the results. Because about this issue, besides a distinctive limit value for AFP and hCG rates in pregnancies to be ended normally and abnormally are not known, there has been studies suggesting that in a population analysed in a rational approach (complication expected) AFP rates must be triply increased rates. Also, in studies which AFP cut off value was taken over 2 MoM for two different serum samples or 2.5 MoM or above just one sample and hCG is over 4 MoM higher predictive rates can be reached, increasing of the limit value accepted and practice with much wider populations, may increase predictivity^(9,31)^.

As a result of our research, in the second trimester unexplained AFP and hCG rates have been found related to adverse perinatal outcomes. Pregnancies in which both AFP and hCG rates increasing together are being more complicated with adverse perinatal outcomes more and in a more serious manner than pregnancies in which rates increase one by one. In the second trimester screening test, without the chromosomal or structural anomaly in a single pregnancy whose pregnancy week calculation is correct, increased AFP and hCG levels must be stimulant in terms of adverse perinatal outcomes, and by starting the follow-up in early period, effort should be made for increasing the fetal survival. Prospective studies are needed for decreasing adverse perinatal outcomes.

## Figures and Tables

**Table 1:**
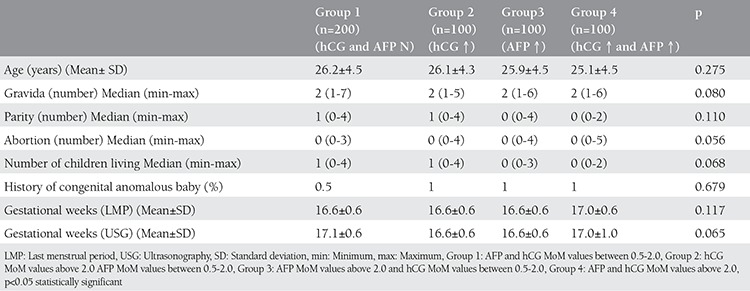
Demographic characteristics of the groups

**Table 2:**
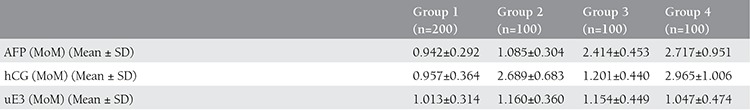
Laboratory data of the groups

**Table 3:**
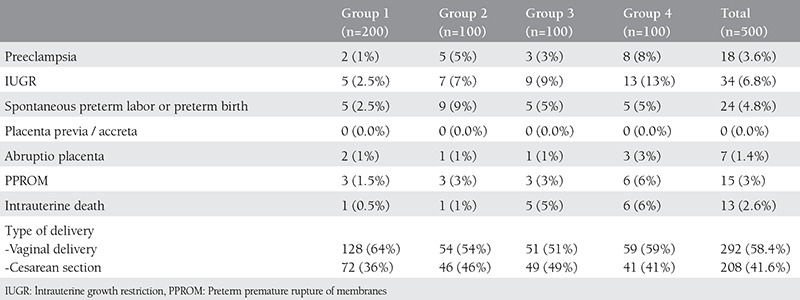
Pregnancy-related complications and type of delivery of the groups

**Table 4:**
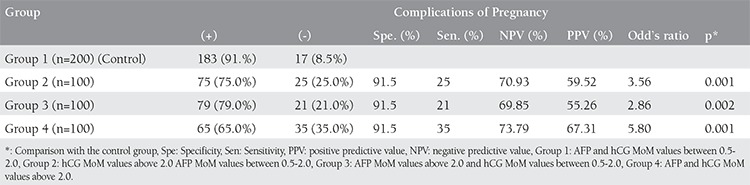
The sensitivity, specificity and predictive rates of serum AFP and hCG levels for predicting pregnancy complications
